# Mental health status, hope and resilient coping in Portuguese higher education students during the COVID-19 pandemic

**DOI:** 10.1192/j.eurpsy.2022.961

**Published:** 2022-09-01

**Authors:** C. Laranjeira, A. Querido

**Affiliations:** Polytechnic of Leiria, School Of Health Sciences/ Citechcare, Leiria, Portugal

**Keywords:** mental health, coping, Pandemics, students

## Abstract

**Introduction:**

The current pandemic crisis disturbed the life of universities and college campuses leading to an overwhelming effect on the educational system, social life, and mental health of students. In this scenario, coping strategies like resilience and hope provide a counterbalance in periods of uncertainty and stress.

**Objectives:**

This study aims to: a) evaluate the prevalence and severity of depression, anxiety, and stress among higher education students during the COVID-19 pandemic; b) characterize the hope and resilient coping levels of graduate students.

**Methods:**

Using a convenience sampling method, online self-reported data were collected between April 2020 to January 2021. The information gathered includes a Sociodemographic Form, the Depression, Anxiety, and Stress Scale (DASS-21) the Brief Resilient Coping Scale (BRCS) and the Herth Hope Index (HHI).

**Results:**

A total of Portuguese 1522 students (75.1% women and 24.9% men) took part in this study. The sample mean age was 22.88±6.93 years [range 18-59 years]. We identified a significant prevalence of symptoms of stress (35.7%), anxiety (36.2%) and depression (28.5%) in our population. The BRSCS score indicated that 60.2% of students exhibited low, 22.7% moderate and 17.1% high levels of resilient coping. The HHI mean was 35.53±5.92 [range 12-48].

**Conclusions:**

The study findings indicate a substantial portion of the students is at high risk of psychological consequences during the COVID-19 pandemic. This study recommends that is needed to get a wider picture of today’s “new normal” education and to develop supportive strategies to enhance students’ mental health and well-being in future pandemics.

**Disclosure:**

No significant relationships.

